# Role of *Staphylococcus aureus* Nasal Carriage in Uremic Pruritus and Infection in Hemodialysis Patients

**DOI:** 10.1111/ijd.17904

**Published:** 2025-06-17

**Authors:** Mei‐Ju Ko, Jenny Chiang, Chun‐Hsing Liao, Wan‐Chuan Tsai, Ping‐Hsiu Tsai, Le‐Yin Hsu, Fang‐Yeh Chu, Yen‐Ling Chiu, Hon‐Yen Wu

**Affiliations:** ^1^ Department of Dermatology Taipei City Hospital Taipei City Taiwan; ^2^ Department of Dermatology National Taiwan University Hospital and College of Medicine Taipei City Taiwan; ^3^ University of Taipei Taipei City Taiwan; ^4^ School of Medicine, College of Medicine National Yang Ming Chiao Tung University Taipei City Taiwan; ^5^ Department of Internal Medicine Far Eastern Memorial Hospital New Taipei City Taiwan; ^6^ Center for General Education and Department of Applied Cosmetology Lee‐Ming Institute of Technology New Taipei City Taiwan; ^7^ Department of Nursing Asia Eastern University of Science and Technology New Taipei City Taiwan; ^8^ Institute of Epidemiology and Preventive Medicine College of Public Health, National Taiwan University Taipei City Taiwan; ^9^ Department of Clinical Pathology Far Eastern Memorial Hospital New Taipei City Taiwan; ^10^ Graduate School of Biotechnology and Bioengineering Yuan Ze University Taoyuan City Taiwan; ^11^ Department of Medical Research Far Eastern Memorial Hospital New Taipei City Taiwan; ^12^ Graduate Institute of Clinical Medicine National Taiwan University Taipei City Taiwan; ^13^ Graduate School of Biomedical Informatics Yuan Ze University Taoyuan City Taiwan; ^14^ Department of Internal Medicine National Taiwan University Hospital and College of Medicine Taipei City Taiwan

**Keywords:** hemodialysis, infection risk, nasal carriage, *Staphylococcus aureus*, uremic pruritus

## Abstract

**Background:**

While *Staphylococcus aureus (S. aureus)* is a well‐recognized concern in hemodialysis (HD) patients, its role in uremic pruritus remains unknown. This study investigated the impact of *S. aureus* nasal carriage on the prevalence and severity of uremic pruritus and its association with subsequent infection in HD patients.

**Methods:**

We conducted a prospective cohort study from April 2019 to January 2020, enrolling 100 HD patients in Taiwan. Nasal swab cultures determined *S. aureus* colonization status. Odds ratios (ORs) for uremic pruritus, effect estimates for pruritus intensity, and hazard ratios (HRs) for subsequent *S. aureus* infections were assessed.

**Results:**

*S. aureus* nasal carriage was detected in 24% of HD patients and was more prevalent among those with uremic pruritus (32.7% vs. 13.3%, *p* = 0.02). *S. aureus* nasal carriage was associated with a significantly increased risk of uremic pruritus (OR: 4.21; 95% confidence interval [CI], 1.34–13.24, *p* < 0.01) and correlated with higher pruritus intensity (effect estimate: 1.18; 95% CI, 0.05–2.30; *p* = 0.04). During a median follow‐up of 8.97 months, five patients developed *S. aureus* infections. While *S. aureus* nasal carriage showed a trend toward an increased risk of subsequent infection, the finding did not reach statistical significance (HR: 50.93; 95% CI, 0.62–4181.02; *p* = 0.08).

**Conclusions:**

*S. aureus* nasal carriage is significantly associated with the prevalence and severity of uremic pruritus in HD patients. Larger studies are needed to confirm its role in infection risk and to explore decolonization as a potential therapeutic strategy.

## Graphical abstract

Schematic representation showing the association between *Staphylococcus aureus* nasal carriage and increased prevalence and severity of uremic pruritus in hemodialysis patients, along with a potential link to subsequent infection risk.

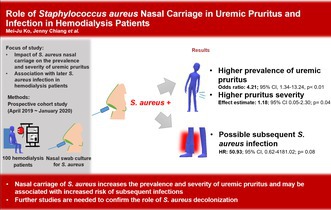

## 1 Introduction

Uremic pruritus is a chronic and distressing symptom experienced by hemodialysis (HD) patients, affecting 38% to 84% of this population [1, 2]. It impacts the quality of life of dialysis patients and is associated with a poor prognosis [3, 4]. The pathophysiology of uremic pruritus remains poorly understood, and current treatment options are limited, highlighting the need for deeper insights into its underlying mechanisms [5].

Emerging evidence suggests that the skin microbiota may play a role in chronic inflammatory skin diseases, potentially offering insights into the pathogenesis of uremic pruritus [6]. For example, atopic dermatitis (AD) is characterized by reduced skin microbial diversity and increased colonization by *Staphylococcus aureus* ( *S. aureus* ) [7], which has been linked to disease flares [8]. Similarly, patients with psoriasis exhibit increased *S. aureus* colonization on the skin and in the nares compared to healthy controls [9], implicating *S. aureus* in the pathogenesis of various skin diseases. Additionally, mechanistic studies have shown that *S. aureus* secretes a protease V8, which activates proteinase‐activated receptor 1 expressed on neurons, driving itch and skin damage in murine models [10]. Despite these findings, the interplay between *S. aureus* and uremic pruritus remains largely unexplored.

In HD patients, the incidence of *S. aureus* nasal carriage is significantly higher compared to healthy controls [11]. In over 90% of HD patients, the *S. aureus* strains isolated from infection sites are identical to those from the anterior nares, suggesting that nasal strains are the likely source of these infections [12]. Given that *S. aureus* colonization generally precedes infection [13], the presence of nasal *S. aureus* in patients with uremic pruritus may not only exacerbate pruritus severity but also increase the risk of subsequent systemic infections.

Our study aimed to elucidate the potential role of *S. aureus* in the pathogenesis of uremic pruritus and assess its impact on infection risk in HD patients. We investigated the association between *S. aureus* nasal carriage and the prevalence and severity of uremic pruritus in a tertiary medical center. Additionally, we evaluated the potential link between *S. aureus* nasal carriage and the risk of subsequent *S. aureus* infections in these patients.

## 2 Materials and Methods

### 2.1 Study Participants

We conducted a prospective cohort study at Far Eastern Memorial Hospital, a tertiary medical center in Taiwan, from April 2019 to January 2020. Adults (≥ 20 years) undergoing maintenance hemodialysis (HD) for more than 3 months were included. Patients were excluded if they had other primary skin disorders causing itching (e.g., urticaria, atopic dermatitis, scabies, or other pruritic dermatoses), ongoing infections at study initiation, or communication difficulties that hindered pruritus assessment. Exclusion decisions were based on medical history and clinical diagnoses by dermatologists or nephrologists at enrollment. The Institutional Review Board of Far Eastern Memorial Hospital approved this study, and all participants provided written informed consent.

### 2.2 Patient Characteristics

Demographic and clinical data, including age, sex, comorbid diseases, cause of end‐stage renal disease, and dialysis details, were recorded. Patients rated the intensity of their pruritus on a visual analogue scale (VAS) scored from 0 to 10 (0 = no pruritus, 10 = worst imaginable pruritus) [14]. Dietary protein intake was assessed using the normalized protein catabolic rate (nPCR) [15]. Dialysis adequacy was assessed by Kt/V, calculated through mathematical modeling based on Daugirdas' method [16].

To examine the status of *S. aureus* nasal carriage, nasal swabs were collected for culture analysis. The nasal swab tests and laboratory examinations were conducted at the central laboratory of Far Eastern Memorial Hospital.

The study participants were prospectively followed until the development of an *S. aureus* infection or until January 31, 2020, whichever occurred first. Participants were censored at the end of follow‐up or at death. The occurrence of *S. aureus* infection was determined based on microbial culture and clinical diagnosis. In cases of culture‐confirmed *S. aureus* growth, a physician differentiated infection from colonization to make the diagnosis.

### 2.3 Statistical Analysis

Data are presented as median (first, third quartiles) or number (percentage). The appropriate statistical tests, including independent sample *t*‐test, Wilcoxon rank‐sum test, chi‐squared test, or Fisher's exact test, were used to compare variable distributions between groups.

Multivariate logistic regression models with stepwise variable selection were used to identify risk factors associated with the presence of uremic pruritus. In addition, multivariate linear regression models with stepwise variable selection were performed to assess the potential predictors of higher pruritus intensity. To analyze the cumulative incidence of *S. aureus* infection among *S. aureus* carriers and noncarriers during follow‐up, we employed Kaplan–Meier survival analysis along with the log‐rank test. Multivariate Cox proportional hazards models with stepwise variable selection were employed to analyze the effect of *S. aureus* nasal carriage on infection risk. A two‐sided *p* value ≤ 0.05 indicated statistical significance. All statistical analyses were performed using SAS (version 9.4, SAS Institute, Cary, NC, USA).

## 3 Results

### 3.1 Patient Characteristics

Among 100 eligible HD patients, 69% were male, with a mean age of 61.7 ± 8.0 years. The average duration of dialysis was 6.7 years. Table 1 displays the demographic characteristics and laboratory data of participants with and without uremic pruritus. Over half (55%) experienced uremic pruritus, with a median VAS score of 4.0 for pruritus intensity. Nasal carriage of *S. aureus* was detected in 24 patients (24%). Among them, methicillin‐resistant *S. aureus* (MRSA) was identified in two patients without pruritus and one patient with pruritus. Data on MRSA were too sparse for further analysis. Patients with pruritus had a higher prevalence of *S. aureus* nasal carriage compared to those without (32.7% vs. 13.3%; *p* = 0.02) and a higher level of serum albumin (4.1 vs. 3.9 g/dL; *p* = 0.02).

**TABLE 1:** Comparison of patient characteristics between hemodialysis patients with and without pruritus.

Variable	With pruritus	Without pruritus	*p*
Number of participants	55	45	
VAS score of pruritus intensity	4.0 (2.1–5.2)	0.0 (0.0–0.0)	< 0.0001**
*S. aureus* nasal carriage	18 (32.7%)	6 (13.3%)	0.02*
Age (years)	63.3 (56.6–66.5)	61.2 (58.0–65.7)	0.35
Female	19 (34.6%)	12 (26.67%)	0.40
Dialysis vintage (years)	5.2 (1.7–10.6)	2.4 (1.0–8.8)	0.10
Kt/V (Daugirdas)	1.3 (1.2–1.4)	1.3 (1.2–1.4)	0.22
White blood cells (1000/μL)	6.3 (4.9–7.3)	6.5 (5.0–8.0)	0.35
Hemoglobin (g/dL)	11.1 (10.6–12.0)	11.3 (10.7–12.0)	0.47
Platelets (1000/μL)	186.0 (138.0–228.0)	205.0 (168.0–247.0)	0.07
Uric acid (mg/dL)	7.4 (6.4–8.5)	7.8 (6.9–8.7)	0.14
Albumin (g/dL)	4.1 (3.8–4.3)	3.9 (3.8–4.1)	0.02*
Aspartate transaminase (U/L)	18.0 (14.0–22.0)	16.0 (14.0–20.0)	0.21
Alanine transaminase (U/L)	15.0 (12.0–18.0)	14.0 (11.0–17.0)	0.13
Alkaline phosphatase (U/L)	94.0 (70.0–122.0)	90.0 (64.0–101.0)	0.07
Total bilirubin (mg/dL)	0.4 (0.3–0.5)	0.4 (0.3–0.5)	0.48
Total cholesterol (mg/dL)	151.0 (134.0–172.0)	149.0 (129.0–184.0)	0.25
Triglyceride (mg/dL)	122.0 (76.0–192.0)	109.0 (85.0–172.0)	0.46
Fasting glucose (mg/dL)	127.0 (90.0–188.0)	121.0 (93.0–164.0)	0.42
Calcium, albumin adjusted (mg/dL)	9.2 (9.0–9.7)	9.2 (8.8–9.6)	0.34
Phosphorus (mg/dL)	4.9 (4.4–6.0)	5.0 (4.2–6.0)	0.48
Ca × P (mg/dL × mg/dL)	47.1 (39.3–54.7)	47.0 (38.7–54.9)	0.48
Ferritin (ng/mL)	307.4 (199.8–436.9)	334.3 (176.0–515.3)	0.22
Intact parathyroid hormone (pg/mL)	186.4 (46.5–411.9)	184.4 (88.1–364.4)	0.32
nPCR	1.2 (1.0–1.4)	1.2 (1.0–1.4)	0.35
Diabetes mellitus	30 (54.6%)	22 (48.9%)	0.57
Cardiovascular diseases	17 (30.9%)	12 (26.7%)	0.64
Cerebrovascular accident	5 (9.1%)	1 (2.2%)	0.22
Hepatitis B	4 (7.3%)	6 (13.3%)	0.34
Hepatitis C	4 (7.27%)	3 (6.67%)	1.00

*Note:* Data are presented as median (first quartile, third quartile) for continuous variables and as number (percentage) for categorical variables. *p* values were calculated using the Wilcoxon rank‐sum test, the *χ* ^2^ test, or Fisher's exact test, as appropriate. *p < 0.05; **p < 0.0001.

Abbreviations: Ca × P, product of albumin‐adjusted serum calcium and serum phosphorus; nPCR, normalized protein catabolic rate; *S. aureus*, *Staphylococcus aureus* ; VAS, visual analogue scale.

### 3.2 *S. aureus* Nasal Carriage and Uremic Pruritus

The multivariate logistic regression analysis showed that *S. aureus* nasal carriage was significantly associated with uremic pruritus in HD patients (odds ratio [OR]: 4.21; 95% confidence interval [CI], 1.38–12.85; *p* = 0.01) (Table 2). The multivariate linear regression analysis showed that *S. aureus* nasal carriage (effect estimate: 1.18; 95% CI, 0.05–2.30; *p* = 0.04) and a higher serum albumin level (effect estimate: 2.16; 95% CI, 0.68–3.64; *p* < 0.01) were independent predictors of higher VAS scores for pruritus intensity (Table 3).

**TABLE 2:** Multivariate logistic regression analysis of predictors for uremic pruritus.

Covariate	Odds ratio	95% confidence interval	*p*
*S. aureus* nasal carriage	4.21	1.38–12.85	0.01
Male	0.50	0.18–1.36	0.17
Age (years)	1.03	0.97–1.09	0.29
Albumin (g/dL)	6.15	1.51–25.07	0.01
Ferritin (ng/mL)	0.998	0.997–1.000	0.08

Abbreviation: *S. aureus*, *Staphylococcus aureus.*

**TABLE 3:** Multivariate linear regression analysis of the predictors of pruritus intensity of uremic pruritus.

Covariate	Parameter estimate	95% confidence interval	*p*
*S. aureus* nasal carriage	1.18	0.05 to 2.30	0.04
Male sex	−0.72	−1.80 to 0.36	0.19
Age (years)	0.04	−0.02 to 0.10	0.19
Cerebrovascular accident	1.72	−0.27 to 3.72	0.09
Albumin (g/dL)	2.16	0.68 to 3.64	< 0.01
Ferritin (ng/mL)	−0.002	−0.003 to 0.0003	0.09

*Note:* Pruritus intensity was assessed using a visual analogue scale score.

Abbreviation: *S. aureus*, *Staphylococcus aureus.*

### 3.3 Risk Factors for *S. aureus* Infection

**FIGURE 1:**
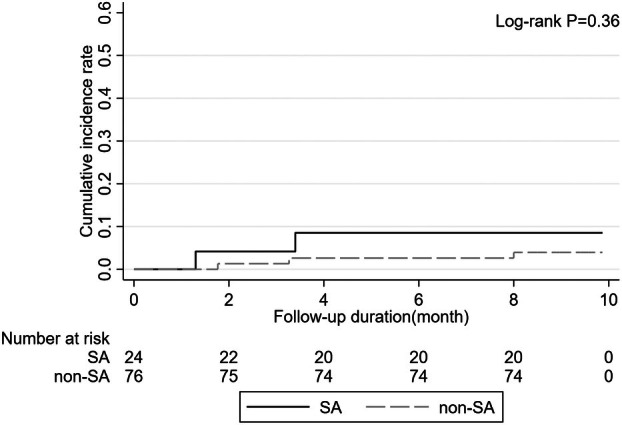
Kaplan–Meier cumulative incidence plot of *Staphylococcus aureus* (SA) infection in carriers and noncarriers.

During a median follow‐up of 8.97 months, five patients developed *S. aureus* infections, including four episodes of cellulitis and one episode of pneumonia. The proportion of patients developing *S. aureus* infection was 7.3% (4/55) among those with uremic pruritus and 2.2% (1/45) among those without uremic pruritus (*p* = 0.25). The Kaplan–Meier cumulative incidence plot did not show a significant difference in the risk of *S. aureus* infection between patients with and without *S. aureus* nasal carriage (*p* = 0.36) (Figure 1). In the multivariate Cox proportional hazards model, *S. aureus* nasal carriage showed a potential association with *S. aureus* infection risk, but this association did not reach statistical significance (hazard ratio [HR]: 50.93; 95% CI, 0.62–4181.02; *p* = 0.08) (Table 4).

**TABLE 4:** Multivariate Cox proportional hazards model of the risk factors for *Staphylococcus aureus* infection.

Covariate	Hazard ratio	95% confidence interval	*p*
*S. aureus* nasal carriage	50.93	(0.62–4181.02)	0.08
VAS score	0.74	(0.31–1.81)	0.51
Male	2.86	(0.03–311.06)	0.66
Age (years)	0.80	(0.64–0.99)	0.04
Cardiovascular diseases	13.75	(0.59–322.77)	0.10
Phosphorus (mg/dL)	0.18	(0.02–1.56)	0.12
Intact parathyroid hormone (pg/mL)	1.01	(1.00–1.02)	0.02
nPCR	0.0004	(0.00–0.73)	0.04

Abbreviations: nPCR, normalized protein catabolic rate; *S. aureus*, *Staphylococcus aureus* ; VAS, visual analogue scale.

## 4 Discussion

This study investigated the relationship between *S. aureus* nasal carriage, uremic pruritus, and the subsequent risk of *S. aureus* infection in HD patients. Our findings revealed a significantly higher prevalence of *S. aureus* nasal carriage among patients with uremic pruritus compared to those without. Furthermore, *S. aureus* nasal carriage was identified as an independent predictor of increased pruritus intensity. However, this association does not establish causality. As this was an observational study, further mechanistic or interventional studies are needed to confirm any causal link.

Several mechanisms could plausibly connect *S. aureus* colonization to uremic pruritus. *S. aureus* superantigens activate T cells, phenol‐soluble modulin α peptide promotes IL‐17 production, and α‐toxin induces mast cell degranulation, all of which can drive skin inflammation and pruritus [17, 18, 19]. Conversely, uremic pruritus may facilitate *S. aureus* colonization through barrier disruption and the elevated skin‐surface pH characteristic of advanced chronic kidney disease (CKD) [20]. This suggests a potentially bidirectional and self‐reinforcing relationship, although direct causality remains to be demonstrated.

While both uremic pruritus and atopic dermatitis exhibit high *S. aureus* colonization rates, the mechanisms may differ. In atopic dermatitis, *S. aureus* colonization is linked to protein‐ and lipid‐related barrier defects and a Th2/Th22‐skewed, adenosine monophosphate (AMP)‐deficient immune profile [21, 22]. In uremic pruritus, it is probably secondary to CKD‐related xerosis, higher skin‐surface pH, and gut microbiota imbalance, with an immune milieu marked by elevated interleukin (IL)‐2, IL‐6, IL‐31, histamine, and altered monocyte subsets [23, 24, 25, 26, 27]. While *S. aureus* is an established contributor to atopic dermatitis, its role in uremic pruritus remains speculative [28]. Given the benefit of anti‐staphylococcal treatments in AD, evaluating nasal decolonization in alleviating uremic pruritus is warranted [29].

In addition to *S. aureus* nasal carriage, higher serum albumin levels were independently associated with uremic pruritus. Although hypoalbuminemia is generally linked to malnutrition and systemic inflammation [30], relatively higher serum albumin levels in our study may reflect better nutritional status and possibly greater ability to communicate pruritus symptoms, or it may arise from selection bias or residual confounding. Previous research shows that HD patients carrying *S. aureus* have a higher infection risk [13]. In our study, the HR for infection among carriers was large but imprecise. The very wide CI and borderline *p* value underscore the substantial statistical uncertainty. A larger, adequately powered cohort study will be required to confirm this association.

This study has several limitations. First, the small sample size, short follow‐up period, and limited number of infection events may have reduced the statistical power of the analysis. Second, only a single nasal swab was performed to assess *S. aureus* colonization, which may have underestimated colonization rates [31]. Repeated sampling at multiple skin sites in future studies would better characterize the temporal dynamics and overall landscape of *S. aureus* carriage. Third, pruritus severity was assessed using the VAS, a widely used and validated tool, which remains inherently subjective. Fourth, because only three MRSA isolates were identified, we could not assess whether methicillin resistance modifies the association between colonization and pruritus or infection risk. Finally, the study was conducted at a single medical center in Taiwan. Therefore, caution is warranted when generalizing our findings to other populations.

In conclusion, our study provides new insights into the interplay between *S. aureus* nasal carriage, uremic pruritus, and infection risk in hemodialysis patients. We identified *S. aureus* nasal colonization as an independent predictor of both the presence and severity of uremic pruritus. While our findings indicate a possible association between *S. aureus* nasal carriage and increased subsequent infection risk, the small sample size and limited number of infection events precluded statistical significance. Larger prospective studies with repeated sampling and extended follow‐up are needed to validate these associations and evaluate the effectiveness of *S. aureus* decolonization in alleviating pruritus among HD patients.

## Conflicts of Interest

The authors declare no conflicts of interest.

## Data Availability

The data that support the findings of this study are available on request from the corresponding author. The data are not publicly available due to privacy or ethical restrictions.
